# Case Report: Three Farmworkers Who Gave Birth to Infants with Birth Defects Closely Grouped in Time and Place—Florida and North Carolina, 2004–2005

**DOI:** 10.1289/ehp.9647

**Published:** 2007-02-21

**Authors:** Geoffrey M. Calvert, Walter A. Alarcon, Ann Chelminski, Mark S. Crowley, Rosanna Barrett, Adolfo Correa, Sheila Higgins, Hugo L. Leon, Jane Correia, Alan Becker, Ruth H. Allen, Elizabeth Evans

**Affiliations:** 1 Division of Surveillance, Hazard Evaluations, and Field Studies, National Institute for Occupational Safety and Health, Centers for Disease Control and Prevention, Cincinnati, Ohio, USA; 2 North Carolina Department of Health and Human Services, Raleigh, North Carolina, USA; 3 Collier County Health Department, Naples, Florida, USA; 4 Florida Department of Health, Tallahassee, Florida, USA; 5 National Center on Birth Defects and Developmental Disabilities, Centers for Disease Control and Prevention, Atlanta, Georgia, USA; 6 Office of Pesticide Programs, U.S. Environmental Protection Agency, Washington, DC, USA

**Keywords:** congenital abnormalities, ectromelia, farmworkers, fungicides, Goldenhar Syndrome, insecticides, micrognathism, pesticides, prevention and control, toxicity

## Abstract

**Context:**

There is little evidence linking adverse reproductive effects to exposure to specific pesticides during pregnancy.

**Case Presentation:**

In February 2005, three infants with congenital anomalies were identified in Collier County, Florida, who were born within 8 weeks of one another and whose mothers worked for the same tomato grower. The mothers worked on the grower’s Florida farms in 2004 before transferring to its North Carolina farms. All three worked during the period of organogenesis in fields recently treated with several pesticides. The Florida and North Carolina farms were inspected by regulatory agencies, and in each state a large number of violations were identified and record fines were levied.

**Discussion:**

Despite the suggestive evidence, a causal link could not be established between pesticide exposures and the birth defects in the three infants. Nonetheless, the prenatal pesticide exposures experienced by the mothers of the three infants is cause for concern. Farmworkers need greater protections against pesticides. These include increased efforts to publicize and comply with both the U.S. Environmental Protections Agency’s Worker Protection Standard and pesticide label requirements, enhanced procedures to ensure pesticide applicator competency, and recommendations to growers to adopt work practices to reduce pesticide exposures.

**Relevance to Professional Practice:**

The findings from this report reinforce the need to reduce pesticide exposures among farmworkers. In addition, they support the need for epidemiologic studies to examine the role of pesticide exposure in the etiology of congenital anomalies.

## Case Reports

In February 2005, the Healthy Start program in the Collier County Health Department (CCHD) in Florida identified three infants with congenital anomalies who were born within 8 weeks of one another and determined that all three mothers had worked for the same tomato grower (CCHD 2005). All three women had worked on the grower’s Florida farms in 2004 before transferring to its North Carolina farms later that year (Table 1). In August 2005, the North Carolina Department of Health and Human Services (NCDHHS) was notified of these births by the North Carolina Department of Agricultural and Consumer Services (NCDACS); this agency had been notified by U.S. Environmental Protection Agency (EPA) Region 4 in April 2005. The Centers for Disease Control and Prevention (CDC) was alerted in September 2005, and with the assistance of state health departments in Florida and North Carolina collected case reports and pesticide exposure histories.

The CCHD searched the Healthy Start program records for other birth defects cases born between December 2004 and February 2005 and whose parents had the potential for exposure to agricultural pesticides. No additional cases were identified. Medical records on the three mothers and their infants were obtained and reviewed by the CCHD and the state health departments in Florida and North Carolina. Charles A. Williams, a clinical geneticist and professor of pediatrics and genetics at the University of Florida, College of Medicine (Gainesville, FL) reviewed case summaries obtained from the medical records and provided the descriptions of the birth defects. Exposure information was obtained from NCDACS and the Florida Department of Agriculture and Consumer Services (FLDACS), which obtained pesticide application and worker assignment records from the grower. Additionally, each of the mothers and fathers were interviewed in early 2005 by CCHD. The mothers of case 1 and case 2 were also interviewed in 2006 by NCDHHS.

Because of the imprecision of the conception date, there is also imprecision in the calendar days that correspond to the maximal sensitivity period for any given birth defect. As the window of maximal sensitivity shifts, the number of days involving pesticide exposure may also change. Therefore, a range of days is provided to reflect the minimum and maximum number of days of pesticide exposure during the maximal sensitivity period. In addition, because workers often worked in several agricultural fields on a given day and because the specific hours worked in each field were not available, days of pesticide exposure were categorized into “probable days” and “possible days.” Probable days are those days when the mother was scheduled to work in a field that had a restricted entry interval (REI) in effect the entire day. Possible days consist of days when the mother was scheduled to work in a field that had an REI that was in effect for only a portion of the day. On possible days, it is conceivable that the mother did not work in the field when the REI was in effect. This would be the case if she worked only before the pesticide application occurred, or only after the REI had expired. According to the Worker Protection Standard (WPS), after the application of any pesticide on an agricultural establishment, the agricultural employer shall not allow or direct any worker to enter or to remain in the treated area before the REI has expired, unless the worker is provided appropriate personal protective equipment (PPE) (U.S. EPA 1997). There is no evidence that PPE was provided to these mothers.

Information on the three cases is provided in Tables 1 and 2. During the period of organogenesis (approximately 14–59 days after fertilization) when birth defects are most likely to occur, all three mothers unknowingly worked in tomato fields that were under an REI because the fields were recently treated with pesticides, some of which have been shown to be teratogenic when tested individually in animals (Table 2). The REIs for the chemicals listed in Table 2 ranged from 12 to 48 hr.

### Case 1

This infant was born with tetra-amelia (absence of all four limbs) (Table 1). The parents had no known birth defect risk factors, and this was the mother’s first pregnancy. The period for limb development is 24–36 days after fertilization (Moore and Persaud 2003). During this period, this child’s mother worked in violation of the REI for up to 4 days involving exposure to several pesticides, including mancozeb (Table 2).

### Case 2

This infant was born with mild Pierre Robin syndrome (micrognathia, high arched palate, and mild persistent palatine rugae). The father of this child has micrognathia. During gestational days (i.e., days after fertilization) 14–57, this child’s mother worked in violation of the REI for up to 8 days. On seven of these days, the pesticides applied to the fields where the mother worked included methamidophos. In addition, on gestational days 7 and 10, the mother worked in fields when an REI was possibly in effect (mancozeb on both days, and abamectin and methylpyrrolidone on day 7). The mother has three other living children, none of whom are known to have birth defects. This mother also had one previous stillbirth but without obvious birth defects.

### Case 3

This infant had multiple severe malformations including cleft lip and palate, imperforate anus, solitary kidney, vertebral anomalies, dysplastic low-set ears, and ambiguous genitalia. These findings are quite reminiscent of a severe type of the Goldenhar Syndrome (also referred to as oculo–auriculo–vertebral sequence). Death occurred at 3 days of age. During gestational days 14–59, the mother worked in violation of REIs for up to 10 days. On eight of these days, the REI for methamidophos was in effect on some of the fields where the mother worked. Abamectin and methylpyrrolidone were applied to some of the fields on two other days, but the mother may have worked in those fields before the applications were made. The mother had two previous pregnancies. One pregnancy 3 years earlier involved a malformed fetus and ended in miscarriage. The mother could not recall her employment or whether she had any toxic exposures during that pregnancy. The other previous pregnancy resulted in a normal child.

None of the three mothers reported tobacco or alcohol use, and none reported taking prescription, over-the-counter, or folk medications. Maternal infections (e.g., syphilis, rubella, cytomegalovirus, toxoplasmosis, and herpes simplex virus) were ruled out as a cause of the birth defects based on data available in the medical records. All three mothers are Mexican, have undocumented U.S. immigrant status (i.e., they did not have a U.S. visa or other immigration document), and sought pre-natal care late in their pregnancies. All three mothers reported morning sickness, but none reported to the crew leader or grower that they were acutely poisoned by pesticides while pregnant. Information on housing conditions during the pregnancies was unavailable. Each child’s father was also employed as a farm-worker for the same grower as the three mothers. Unfortunately, information on the three fathers’ pesticide exposures was unavailable.

Approximately 956 farmworkers were employed at the Florida location and 500 at the North Carolina location, 20% of whom were women. The identity of the female farm-workers was not provided by the employer. Thus, identification of other workers who gave birth in 2004 or 2005 was not possible.

In 2005, the Florida and North Carolina farms were inspected by FLDACS and NCDACS, respectively. A large number of violations were identified, and the grower received among the largest fines ever imposed by these enforcement agencies. Violations identified by both agencies included failure to prevent workers from entering pesticide-treated fields before REI expiration, and failure of pesticide handlers to understand all pesticide label requirements. NCDACS also documented failure to provide drinking water and water for routine washing, whereas FLDACS did not report on the availability of water.

## Discussion

Three farmworkers giving birth to infants with birth defects within an 8-week period is cause for concern. In Florida, approximately 3% of live births have major birth defects. There is evidence to suggest that the three observed major birth defects exceed this expected rate. To obtain the observed birth defects rate among these farmworkers, one needs the number of births for the period in question. Unfortunately, there is no accurate information on the fertility rate among female farm-workers employed in Florida. However, based on Collier County, Florida, Hispanic birth rates (Florida Department of Health 2001; U.S. Census Bureau 2001), it is estimated that 25 live births occur every year among the 191 female farmworkers employed in Florida where the case mothers worked, or two live births per month. Assuming the other three births were normal (of the six expected live births in the 12-week period that was investigated), these three infants with birth defects would provide an incidence rate of 50% for birth defects during the 12 weeks in question among the female farmworkers employed in Florida where the case mothers worked.

The etiology of most human birth defects cases is unknown (Moore and Persaud 2003). However, multifactorial interactions of genetic and environmental factors are thought to be responsible for 20–25% of birth defects, genetic factors alone for 15–25%, and environmental agents alone for 7–10% (Moore and Persaud 2003). Known risk factors include chromosomal disorders, single gene mutations, family history of birth defects, alcohol, some medications, infections, tobacco, diabetes, and lack of essential vitamins (e.g., folic acid) (CDC 2006). Although animal toxicologic studies provide evidence that high doses of some pesticides can alter reproductive function and produce birth defects, few epidemiologic studies have linked specific pesticide exposures to reproductive toxicity in humans (Hanke and Jurewicz 2004; Sever et al. 1997; Thulstrup and Bonde 2006).

There are serious concerns that during the period of organogenesis all three mothers were exposed early in pregnancy to pesticides shown to be teratogenic in animals. Furthermore, some of these exposures may have been high because, according to the grower’s records, the mothers worked in fields in which the REI had not expired.

Mancozeb and its metabolite ethylenethiourea (ETU) have been shown to produce limb defects and cleft palate after high oral doses were given to rats (Larsson et al. 1976). ETU has a biologic half-life of approximately 34 hr to 4 days (Kurttio and Savolainen 1990). During the period of limb development, the mother of case 1 may have worked up to 4 days in fields in violation of the REI for this fungicide.

The mother of case 3 has evidence of 8 workdays (4 probable and 4 possible days) of exposure to methamidophos, which has evidence for teratogenicity in mice and rats (Asmatullah and Aslam 1999; Hanafy et al. 1986). On at least three of the probable days, this mother may have been exposed within 14 hr of the application. The REI for methamidophos is 48 hr. However, the U.S. EPA recommended in 2002 that the REI be increased to 96 hr (U.S. EPA 2002). If the 96-hr REI had been in effect in 2004, then this mother would have had 10 days of working in violation of the REI [8 probable (days 22–24, 27, 30, 31, 33, 34), and 2 possible (days 26 and 27)]. Despite some animal evidence for teratogencity, we are aware of no authoritative sources, including the U.S. EPA and the State of California Environmental Protection Agency, that have concluded that methamidophos is a teratogen (FLDACS 2005; Food and Agriculture Organization/World Health Organization 2002).

The mother of case 2 had only one probable day working in violation of an REI during the maximal sensitivity period, the fewest number of the three mothers. However, this mother had 8 days possibly involving work in violation of an REI. The one probable day and six of the possible days involved exposure to methamidophos. We are unaware of animal evidence for an association between the birth defects found in case 2 and methamidophos exposure; however, mancozeb and its metabolite ETU have been shown to produce abnormal shortening of the mandible (Larsson et al. 1976; Stula and Krauss 1977). Although the potential mancozeb exposure for the mother of case 2 was on days 7 and 10, the half-life of this chemical and its metabolite suggests the possibility of exposure during organogenesis.

Some may question whether this is a true cluster because each of the babies had a different constellation of defects, and none of the pesticides to which the mothers were probably exposed can be linked (in animal or human studies) to all of the observed defects. However, the differences in the birth defects identified in this cluster may be attributed to the differences in the conception dates, the differences in the work histories of the three mothers, and the large number of chemicals used by the grower. There is evidence to suggest that each mother was exposed to pesticides during the maximal sensitivity period for the organ system/structure that was affected.

As demonstrated in Table 2, all mothers had the potential for exposure to pesticide mixtures, and little is known about the teratogenicity of these mixtures. Animal toxicologic studies are performed on individual chemicals, and little is known about the reproductive effects of exposure to mixtures of pesticides that have different modes of action. However, there is evidence in a mice model that pesticide mixtures can produce developmental effects that would not be predicted or are more severe than predicted based on the known toxicology of each individual pesticide (Cory-Slechta 2005).

All three mothers had the potential for three routes of exposure: dermal, inhalation, and oral. It is not possible to determine which route most contributed to their internal dose of pesticides. Many host, agent, and environmental factors affect the relationship between the potential exposure and the resulting absorbed dose (Solomon et al. 2005). When the route of exposure is dermal, the internal dose can be modified by many factors including the area and location of the skin exposed, the amount of pesticide residue on the foliage that can be dislodged, the presence of skin damage, environmental temperature and humidity, the presence of other compounds on the skin, and the inherent transcutaneous absorption properties of the pesticide (Boeniger 2003). As for the inhalational route of exposure, the internal dose can be modified by the respiratory rate, which increases with physical exertion, and the vapor pressure of the pesticide. Finally, oral exposure can take place if pesticide-contaminated food or drink was consumed. This could occur if the mothers did not wash their hands before eating. Among the citations issued by NCDACS was a lack of hand-washing facilities at one farm site where the women worked, and failure to provide adequate amounts of drinking water (Chelminski and Higgins 2006). In addition, there is no evidence that the women were provided with PPE, such as chemically resistant gloves and clothing to reduce dermal exposure to pesticides.

There is no evidence that the three mothers described in this report experienced toxicity associated with their gestational pesticide exposures. Although many teratogenic exposures also produce adverse effects on the mother, there are exceptions. For example, a study in which rats were administered a dermal dose of 50 mg/kg body weight/day of ETU on days 12 and 13 of gestation produced malformations in all fetuses (including encephalocele, short mandible, and missing leg bones) but produced no substantial acute effects on the dams (FLDACS 2005; Stula and Krauss 1977). Another study in which pregnant mice were given a single intrapertioneal injection of 80 mg/kg body weight of chlorpyrifos (an organophosphate pesticide) produced a significantly increased rate of malformed fetuses, including cleft palate and absent thoracic vertebrae, compared to a control group exposed only to the vehicle, but the pesticide produced no overt signs of maternal toxicity (Tian et al. 2005). The dose of 80 mg/kg body weight of chlorpyrifos was chosen by the investigators because it was “below doses that may cause significant inhibition of cholinesterase activity” (Tian et al. 2005). Cholinesterase inhibition is one of the most sensitive toxic end points produced by organophosphate pesticides (U.S. EPA 2006a). In addition, newborn children, and perhaps fetuses, may be substantially more susceptible to toxicity from pesticide exposure compared to their mothers. For example, plasma paraoxonase 1, an enzyme involved in organophosphate detoxification, has recently been shown to have both significantly lower concentrations and significantly lower enzyme detoxification activity in newborn infants compared with their mothers, suggesting increased susceptibility to organophosphate toxicity among infants (Furlong et al. 2006; Holland et al. 2006). With increased susceptibility, pesticide exposure may produce toxicity in the fetus while sparing the mother.

Although evidence of acute pesticide poisoning among the three mothers is absent, limited contemporaneous means were available to identify maternal pesticide toxicity. Their undocumented immigrant status and lack of health insurance limited their access to medical care, as evidenced by the fact that none of the three mothers received prenatal care before the second trimester of pregnancy. Furthermore, because the mothers may not have known the symptoms associated with pesticide toxicity, any such symptoms may have been attributed to their pregnancy. All three mothers reported morning sickness, whose symptoms such as nausea and headache can resemble pesticide poisoning.

There are several limitations with this report. Because a complete cohort of the grower’s employees could not be ascertained, it was not possible to fully characterize their birth defect risk. Because some birth defects are not diagnosed for months to years after birth, it is possible that additional undetected birth defects exist among this farmworker cohort. Although the presence of family history for case 2 and the multiple, complex defects for case 3 suggest the likelihood of a genetic etiology, it was not possible to conduct evaluations of genetic causes. Information on pesticide exposure was based on company records, which may be inaccurate. Because pesticide biomonitoring and environmental sampling were not performed, the mothers’ exposures could not be compared with the high doses used in animal testing to produce developmental effects. In addition, all three mothers received late prenatal care, and nutritional supplementation was not begun until after prenatal care commenced. Moreover, previous reports of clusters of birth defects and presumed occupational exposures have, in light of additional evidence, been found to be more complicated problems or related to factors not measured in the original studies (Missmer et al. 2006; Williams et al. 2002). Finally, information was unavailable on paternal occupational pesticide exposures, although each child’s father was also employed as a farmworker for the same grower as the three mothers. Given these limitations, the small number of cases, the lack of an epidemiologic study involving the grower’s cohort of exposed workers in Florida and North Carolina, and no known published epidemiologic studies of birth defects and the pesticides of concern, the evidence available is inadequate to establish a causal relationship with pesticide exposures.

Despite these limitations and the lack of a clear etiology for the observed birth defects, the case series raises serious concerns that some farmworkers may experience unsafe pesticide exposures when pesticide label directions are not followed (U.S. EPA 1996). These exposures reinforce the importance of compliance with and enforcement of existing pesticide regulations, including the WPS and the Occupational Safety and Health Administration’s Field Sanitation Standard (1987). North Carolina and Florida have approximately 54,000 and 44,000 farms respectively (U.S. Department of Agriculture 2004), but only 23 and 20 farm inspectors to enforce pesticide regulations (U.S. EPA 2006b). Strengthened procedures to certify the competency of private pesticide applicators for safe pesticide applications also may be needed. In addition, work practices should be implemented to reduce pesticide exposures. In late 2005, the grower voluntarily agreed to cease use of mancozeb, methamidophos, and abamectin. It is important that appropriate training be provided to farmworkers, including information on the adverse effects associated with occupational pesticide exposures. In addition, because all three mothers sought prenatal care only late into their pregnancy, improved access to medical care among farmworkers appears needed. Finally, improved surveillance programs for pesticide-related illness and birth defects are needed, as well as increased capacity to investigate future birth defects clusters with suspected workplace etiologies.

## Figures and Tables

**Table 1:** Demographic and work history information on the infants with birth defects and their mothers.

Birth defects	Date of birth	Estimated date of conception^a^	Sex	Age of mother at time of infant’s birth (years)	Days mother was employed on grower’s Florida farms after conception^b^	Days mother was employed on grower’s North Carolina farms after conception^b^
Case 1						
Tetra-amelia	17 Dec 2004	3 Apr 2004	Male	19	0–14	16–182
Case 2						
Micrognathia (underdeveloped jaw), high arched palate, and mild persistent palatine rugae	4 Feb 2005	10 Apr 2004	Male	30	0–51	65–216
Case 3						
Multiple malformations including cleft lip and palate, imperforate anus, solitary kidney, vertebral anomalies and very abnormal, dysplastic, low-set ears, and ambiguous genitalia, reminiscent of a severe type of Goldenhar syndrome	6 Feb 2005	16 May 2004	Female	21	20–36	120–159

a The conception date was calculated by adding 14 days to the onset date of the last menstrual period. Because the precise date of conception was unavailable, this date represents the first date in a 2- week window that is thought to capture the precise conception date.

b Based on the conception date provided in this table.

**Table 2:** Days worked during the first 2 months of pregnancy in violation of an REI and pesticides for which an REI was in effect.

				Specific gestational days worked in violation of an REI to the given pesticide^b^		
Mother	Estimated period of organogenesis for specific birth defect (days after fertilization)^a^	Total days worked in specified period of organogenesis^b^	Pesticides mother was potentially exposed to during maximal sensitivity period	Specific probable days^c^	Specific possible days^d^	Some teratogenic findings from testing of pesticide in animals^e^
Case 1	24–36	2–6	Mancozeb	Days 19, 32, 37, 39, 41		Limb reduction defects, cleft palate, and brachygnathia
			Copper hydroxide	Days 19, 32, 37, 39, 41		No data found
			*Bacillus thuringiensis*	Days 19, 32, 37, 41		No data found
			Spinosad	Days 31, 39		No teratogenicity identified
			Azadirachtin	Day 41		No data found
			*Bacillus subtilis*	Day 41		No data found
Case 2	14–57	21–27	Mancozeb		Days 7, 10	Limb reduction defects, cleft palate and brachygnathia
			Methamidophos	Days 10, 28	Days 7, 26, 27, 38, 39, 40	Anotia, anencephaly, paddle-shaped limbs, microphthalmia
			Abamectin		Day 7	Cleft palate
			Methylpyrrolidone^f^		Day 7	Cleft palate
			Copper hydroxide		Days 7, 10, 45	No data found
			Fenpropathrin		Days 7, 10, 26, 27, 28, 38, 39	No teratogenicity identified
			Chlorothalonil		Days 10, 26, 27, 28, 38, 39	No teratogenicity identified
			Esfenvalerate		Day 7	No teratogenicity identified
			Methomyl		Day 45	No teratogenicity identified
Case 3	14–59	5–11	Methamidophos	Days 22, 30, 33, 34	Days 23, 25, 26, 31	Anotia, anencephaly, paddle-shaped limbs, microphthalmia
			Abamectin		Days 24, 29	Cleft palate
			Methylpyrrolidone^f^		Days 24, 29	Cleft palate
			Fenpropathrin	Days 30, 33	Days 22, 24	No teratogenicity identified
			Hydrogen dioxide		Day 23	No data found
			Chlorothalonil		Day 24	No teratogenicity identified

a Based on Moore and Persaud (2003).

b Because of the imprecision of the conception date, there is also imprecision in the calendar days that correspond to the maximal sensitivity period. As the window of maximal sensitivity shifts, the number of qualifying days may change. The numbers provided reflect the range of qualifying days.

c Probable days are those days when the worker was scheduled to work in a field that had an REI that was in effect the entire day.

d Possible days consist of days when the mother worked in a field that had an REI that was in effect for only a portion of the day. On possible days, it is conceivable that the mother did not work in the field when the REI was in effect. This would be the case if she worked only before the application occurred, or only after the REI had expired. Detailed information on the hours worked in specific fields was not available.

e As summarized in FLDACS (2005). “No data found” = no studies that explored the teratogenicity of the compound were identified by FLDACS. “No teratogenicity identified” = when teratogenicity studies were conducted and all were found to be negative.

f This chemical is included in the same pesticide product as abamectin but is not an active ingredient (i.e., it is considered an inert ingredient).

## References

[b1-ehp0115-000787] Asmatullah, Aslam T (1999). Toxicity of methamidophos in pregnant mice and developing fetuses. Punjab Univ J Zool.

[b2-ehp0115-000787] Boeniger MF (2003). The significance of skin exposure. Ann Occup Hyg.

[b3-ehp0115-000787] CCHD 2005. Collier County Health Department Investigation into the Occurrence of Congenital Malformations in Immokalee, Collier County, Florida, 2005. Naples, FL:Collier County Health Department, Florida Department of Health. Available: http://www.doh.state.fl.us/chdcollier/pdf/Birth_Defects_Report.pdf [accessed 18 January 2007].

[b4-ehp0115-000787] CDC 2006. Recommendations to improve preconception health and health care—United States: a report of the CDC/ATSDR Preconception Care Work Group and the Select Panel on Preconception Care. MMWR 55(RR-6):1–23. Available: http://www.cdc.gov/mmwr/preview/mmwrhtml/rr5506a1.htm [accessed 18 January 2007]. 16617292

[b5-ehp0115-000787] Chelminski AN Higgins S 2006. Assessment of Maternal Occupational Pesticide Exposures during Pregnancy and Three Children with Birth Defects: North Carolina, 2004. Raleigh, NC:North Carolina Department of Health and Human Services. Available: http://www.epi.state.nc.us/epi/oii/Agmartreleasereport.pdf [accessed 18 January 2007].

[b6-ehp0115-000787] Cory-Slechta DA (2005). Studying toxicants as single chemicals: does this strategy adequately identify neurotoxic risk?. Neurotoxicology.

[b7-ehp0115-000787] FDOH 2001. Florida 2000 Vital Statistics Annual Report. Tallahassee, FL:Florida Department of Health. Available: http://www.flpublichealth.com/VSBOOK/VSBOOK.aspx# [accessed 18 January 2007].

[b8-ehp0115-000787] FLDACS 2005. Teratogenic Potential of Pesticides Associated with Florida Ag-Mart Farmworker Investigation. Tallahassee, FL:Florida Department of Agriculture and Consumer Services.

[b9-ehp0115-000787] Food and Agriculture Organization/World Health Organization 2002. Methamidophos. Pesticide Residues in Food-2002-Joint FAO/WHO Meeting on Pesticide Residues. Available: http://www.inchem.org/documents/jmpr/jmpmono/2002pr10.htm#1.2.5.2 [accessed 18 January 2007].

[b10-ehp0115-000787] Furlong CE, Holland N, Richter RJ, Bradman A, Ho A, Eskenazi B (2006). PON1 status of farmworker mothers and children as a predictor of organophosphate sensitivity. Pharmacogenet Genomics.

[b11-ehp0115-000787] Hanafy MSM, Atta AH, Hashim MM (1986). Studies on the teratogenic effects of tamaron (an organophosphorus pesticide). Vet Med J.

[b12-ehp0115-000787] Hanke W, Jurewicz J (2004). The risk of adverse reproductive and developmental disorders due to occupational pesticide exposure: an overview of current epidemiological evidence. Int J Occup Med Environ Health.

[b13-ehp0115-000787] Holland N, Furlong C, Bastaki M, Richter R, Bradman A, Huen K (2006). Paraoxonase polymorphisms, haplotypes, and enzyme activity in Latino mothers and newborns. Environ Health Perspect.

[b14-ehp0115-000787] Kurttio P, Savolainen K (1990). Ethylenethiourea in air and in urine as an indicator of exposure to ethylenebisdithiocarbamate fungicides. Scand J Work Environ Health.

[b15-ehp0115-000787] Larsson KS, Arnander C, Cekanova E, Kjellberg M (1976). Studies of teratogenic effects of dithiocarbamates maneb, mancozeb, and propineb. Teratology.

[b16-ehp0115-000787] Missmer SA, Suarez L, Felkner M, Wang E, Merrill AH, Rothman KJ (2006). Exposure to fumonisins and the occurrence of neural tube defects along the Texas–Mexico border. Environ Health Perspect.

[b17-ehp0115-000787] Moore KL Persaud TVN 2003. The Developing Human: Clinically Oriented Embryology. 7th ed. Philadelphia:W.B. Saunders.

[b18-ehp0115-000787] Occupational Safety and Health Administration 1987. Field Sanitation Standard. 29 CFR 1928.110. Available: http://www.osha.gov/pls/oshaweb/owadisp.show_document?p_table=STANDARDS&p_id=10959 [accessed 18 January 2007].

[b19-ehp0115-000787] Sever LE, Arbuckle TE, Sweeney A (1997). Reproductive and developmental effects of occupational pesticide exposure: the epidemiologic evidence. Occup Med.

[b20-ehp0115-000787] Solomon KR, Houghton D, Harris SA (2005). Nonagricultural and residential exposues to pesticides. Scand J Work Environ Health.

[b21-ehp0115-000787] Stula EF, Krauss WC (1977). Embryotoxicity in rats and rabbits from cutaneous application of amide-type solvents and substituted ureas. Toxicol Appl Pharmacol.

[b22-ehp0115-000787] Thulstrup AM, Bonde JP (2006). Maternal occupational exposure and risk of specific birth defects. Occup Med (Lond).

[b23-ehp0115-000787] Tian Y, Ishikawa H, Yamaguchi T, Yamauchi T, Yokoyama K (2005). Teratogenicity and developmental toxicity of chlorpyrifos. Maternal exposure during organogenesis in mice. Reprod Toxicol.

[b24-ehp0115-000787] U.S. Census Bureau 2001. Census 2000 Summary File 1 (Collier County, FL). Washington DC:U.S. Census Bureau. Available: http://factfinder.census.gov/servlet/DatasetMainPageServlet?_program=DEC&_submenuId=datasets_1&_lang=en&_ts= [accessed 18 January 2007].

[b25-ehp0115-000787] U.S. Department of Agriculture. 2004 2002 Census of Agriculture. Washington DC:National Agricultural Statistics Service, U.S. Department of Agriculture. Available: http://www.nass.usda.gov/index.asp [accessed 18 January 2007].

[b26-ehp0115-000787] U.S. EPA 1996. Office of Prevention, Pesticides and Toxic Substances Harmonized Test Guidelines. Occupational and Residential Exposure Test Guidelines. Series 875. Washington, DC:U.S. Environmental Protection Agency. Available: http://www.epa.gov/opptsfrs/publications/OPPTS_Harmonized/875_Occupational_and_Residential_Exposure_Test_Guidelines/index.html [accessed 18 January 2007].

[b27-ehp0115-000787] U.S. EPA 1997. Worker Protection Standard. Washington DC:U.S. Environmental Protection Agency. Available: http://www.epa.gov/pesticides/safety/workers/PART170.htm [accessed 18 January 2007].

[b28-ehp0115-000787] U.S. EPA. 2002. Interim Reregistration Eligibility Decision for Methamidophos. Case No 0043. Washington DC:U.S. Environmental Protection Agency. Available: http://www.epa.gov/oppsrrd1/REDs/methamidophos_ired.pdf [accessed 18 January 2007].

[b29-ehp0115-000787] U.S. EPA 2006a. Organophosphate pesticide (OP) cumulative assessment—2006 Update. Washington DC:U.S. Environmental Protection Agency. Available: http://www.epa.gov/pesticides/cumulative/2006-op/index.htm [accessed 18 January 2007].

[b30-ehp0115-000787] U.S. EPA 2006b. Certification and Training Plan and Annual Report. Pullman, WA:Washington State University. Available: http://134.121.87.199/candt/logon.cfm [accessed 18 January 2007].

[b31-ehp0115-000787] Williams LJ, Honein MA, Rasmussen SA (2002). Methods for a public health response to birth defects clusters. Teratology.

